# Editorial: Innate Cells in the Pathogenesis of Food Allergy

**DOI:** 10.3389/fimmu.2021.709991

**Published:** 2021-06-10

**Authors:** Ana Olivera, Karen Laky, Simon Patrick Hogan, Pamela Frischmeyer-Guerrerio

**Affiliations:** ^1^ Mast Cell Biology Section, Laboratory of Allergic Diseases, National Institute of Allergy and Infectious Diseases, National Institutes of Health (NIH), Bethesda, MD, United States; ^2^ Food Allergy Research Section, Laboratory of Allergic Diseases, National Institute of Allergy and Infectious Diseases, National Institutes of Health (NIH), Bethesda, MD, United States; ^3^ Mary H Weiser Food Allergy Center, Department of Pathology, Michigan Medicine, University of Michigan, Ann Arbor, MI, United States

**Keywords:** food allergy, eosinophilic esophagitis, innate cells, sensitization, cofactors, oral tolerance, microbiota

Food allergy results from a failure to establish or maintain oral tolerance to certain food proteins and is manifested by adverse responses following ingestion of these foods that range from mild to life-threatening. The increasing prevalence in pediatric and adult food allergy in recent decades and the potentially life-threatening nature of reactions make food allergy a significant public health concern (1). Clinical data and experimental studies establish a role for the adaptive immune system, particularly food-specific IgE, in food allergy. Emerging evidence indicates that innate cells, non-adaptive immune processes, and environmental factors including diet, drugs, and exercise also contribute to abnormal responses to food (2, 3). However, precisely how innate cells, non-adaptive immune processes and factors impact the food allergy diathesis remains poorly understood.

This Frontiers Research Topic represents a collection of articles from worldwide experts that collectively present a current overview of mechanisms in food allergy, diagnostics, and treatments, with particular emphasis on the roles played by innate immune cells. We received a total of 16 articles, including 8 Reviews and 1 Perspective on central topics to food allergy and eosinophilic esophagitis (EoE); and 7 Original Research articles that describe various mechanistic aspects and involvement of innate cells, mediators, microbiota, and factors that alter responses to food in mouse models and humans.

## Microbiota, Passage of Antigens and Th2 Responses in the Sensitization to Food Antigens

The intestinal epithelium regulates passage of molecules across the gut wall to the underlying immune compartment and is critical for homeostatic tolerance to food and microbial antigens (3). A tolerogenic response is thought to be primarily mediated by antigen specific, peripherally derived CD4^+^FoxP3^+^ T regulatory cells (Tregs) which are fortified by additional supportive regulatory mechanisms including MHCII^+^CX3CR1^Hi^IL-10 producing macrophages and commensal microbes and their metabolites (4). Ali et al. discuss how damage to or alteration of intestinal epithelial cells compromises active tolerogenic processes that normally limit innate immune cell induced type 2 (Th2) immune responses and oral antigen sensitization. Furthermore, they discuss the cellular and molecular mechanisms by which food and microbial antigens cross the intestinal epithelial barrier to activate innate immune pathways and immune tolerance mechanisms. Much of our current understanding of mechanisms responsible for oral tolerance and food sensitization have been derived from animal-based studies. Bruton et al. discuss the advantages and challenges of models of food allergy to study tolerance and sensitization mechanisms and summarize evidence supporting the prominent roles of innate cell types in the elicitation of allergic sensitization.

Smeekens et al. demonstrate the importance of intestinal epithelial barrier in preventing food sensitization. The authors describe a mouse strain (CC027/GeniUnc) genetically susceptible to food allergy, in the absence of adjuvants. These mice had reduced fecal IgA and alterations in certain bacterial phyla upon exposure to antigens, which promoted increased antigen absorption, and this was associated with increased food-specific IgE. Mennini et al. also highlight a possible role for the microbiota-innate immune axis in food sensitization describing increased bacterial load and microbial dysbiosis in the esophagus of patients with EoE, although a causal relationship remains to be established.

A study by Noah et al. demonstrated that antigen passage across the intestinal epithelium results in sensitization and allergy to food. They show that housing mice at thermoneutrality (26–30°C), a temperature of metabolic homeostasis, enhanced food allergy responses and this was associated with a switch in the mechanism of passage of luminal antigens across the small intestine epithelium from goblet cell antigen passages to secretory antigen passages (SAPs). In further support of the concept that SAPs promote oral food sensitization, a similar process favored development of food allergy in *Il4ra* ^F709^ mice housed at standard temperatures. An elegant study by Farazuddin et al. describes the development and utilization of a nanoscale oil-in-water emulsion vaccine to suppress food allergy. The vaccine induced long-lasting suppression of oral allergen-induced anaphylaxis. Protection was associated with strong IFNγ-mediated suppression of Th2-cytokines, alarmins and ILC2. Intriguingly, the Smeekens and Farazuddin studies also point toward the existence of mechanisms that dissociate antigen-specific IgE from food-induced anaphylaxis: in CC027/GeniUnc mice, elevated IgE to some food allergens, unlike IgE to peanut and walnut, did not trigger anaphylaxis (Smeekens et al.); furthermore, the suppressive effects of the vaccine were achieved despite a persistence of allergen-specific IgE (Farazuddin et al.). Further investigation is warranted to elucidate these regulatory mechanisms.

## Antigen Presenting Cells, Tregs, Monocytes and Eosinophils in Food Allergy

Several articles in this Research Topic underscore the contributions of various immune cells to food allergy following alterations in barrier permeability, as well as the importance of early life “immune education”. Key players in the regulation of oral tolerance versus allergy are antigen-presenting cells (APC), including dendritic cells, monocytes and macrophages. APC present food antigens to CD4^+^ T cells and depending on tissue cues either drive expansion of Th2 cells and promote IgE-specific B cell responses, or expansion of Tregs and tolerance. Based on human and animal model data, Liu et al. review evidence supporting a role for APC populations in the regulation of oral tolerance or sensitization to foods.

Microbiota drive the differentiation of a population of RORγt^+^ Tregs in the gut that is crucial for tolerogenic homeostasis in early life (5). Notably, these Treg populations are reduced in children with food allergies (6). Knoop et al. demonstrate that RORγt^+^ Tregs developed at weaning, but not before, are long-lived and required to suppress Th2 responses and maintain tolerance to antigens later in life. This study supports the importance of immune education early in life for the proper expansion of this population of peri-weaning Tregs that cannot be substituted by post-weaning T cells.

Further supporting a role for dysregulation of early life immune education in food allergy, Neeland et al. demonstrate that infants with food allergy present an altered innate immune signature characterized by increased frequency of a monocytic population that is hyper-responsive to endotoxin stimulation and posit that in early life aberrant reprograming of innate cells is associated with the development of food allergy.

Eosinophils are an innate immune cell lineage that define EoE and are associated with food allergy. Doyle et al. review literature on the potential roles of eosinophils in EoE and food allergy. Based on cumulative observations, they propose a dual role for eosinophils in these disorders where eosinophils 1) are initially protective and actively regulate local tissue immunity and/or 2) if persistently activated drive tissue remodeling and fibrosis.

## The Role of Basophils and Mast Cells

Basophils and mast cells often take the center stage in food allergy as predominant effector cells in IgE-mediated responses. In addition, their rapid responses to bacterial products and mediators released by damaged epithelium position them at the crossroads between the innate and adaptive arms of the immune system, as innate stimulation can alter their responses to antibody cross-linking (7–9). Several articles in this Research Topic summarize critical knowledge on aspects of basophil/mast cell activation, roles in food allergy, utility as diagnostic tools, and targets for pharmacological intervention. Paranjape et al. review the associations of basophil responsiveness and surface marker expression with clinical outcomes in food allergy and blood levels of specific IgG and IgE antibodies in humans. These associations have made ex-vivo basophil activation tests a useful diagnostic tool for diagnosing food allergy and for monitoring progress during immunotherapy. Kanagaratham et al. discuss how IgE and IgG, acting through activating or inhibitory receptors on mast cells, basophils, and other innate cells, regulate acute effector cell reactions and the induction of Th2-immunity in food allergy. Knowledge on the mechanisms involved in such regulation by Ig receptors can be harnessed for therapeutic applications. Complementary to this review, Dispenza et al. summarize therapeutic strategies to target IgE and IgE-receptor signaling in food-induced anaphylaxis.

The role played by gastrointestinal mast cells, which are located in proximity with IgE^+^ plasma cells, in the manifestations of food allergies is discussed by Paranjape et al. and Kanagaratham et al. Ptaschinski et al. implicate soluble stem cell factor (sSCF) in the accumulation of small intestine mast cells in a food allergy model. They show that neutralization of sSCF diminished intestinal mast cell numbers and mediator release after challenge. Reduced mast cell frequency and activation correlated with reduced gut permeability and manifestation of food anaphylaxis reactions. Krajewski et al. show that food-derived components such as curcumin, known for its anti-allergic and anti-inflammatory properties, suppressed mast cell activation and survival by inhibiting protein disulfide isomerase (PDI), a thiol reductase expressed on the surface of mast cells. In a food allergy model, blockade of PDI reduced small intestine mast cell numbers and activation and attenuated food allergy reactions. Both studies add to our understanding of how intestinal mast cells can be regulated and provide rationale for novel treatments for food allergy.

The severity of an allergic reaction to food, even to a particular food allergen in the same individual, can vary substantially for reasons that are poorly understood. Muñoz-Cano et al., review evidence that implicates exogenous cofactors such as non-steroidal anti-inflammatory drugs, exercise, and alcohol, as contributors to this heterogeneity. Mechanisms by which these exogenous factors may potentiate allergic responses by enhancement of mast cell and basophil activation are discussed.

Taken together, this Research Topic on the role of innate cells in the pathogenesis of food allergy provides a valuable collection that gives insight into the many exciting avenues of research that continue to enhance our understanding of food allergy and EoE.

## Author Contributions

All authors contributed to the article and approved the submitted version.

## Funding

This work was supported by the Intramural Research Program of the National Institutes of Health (NIH), National Institute of Allergy and Infectious Diseases (NIAID) (AO, KL and PF-G) and by the National Institutes of Health grants; AI138177, AI140133, AI112626; the Mary H. Weiser Food Allergy Center (MHWFAC) and the Ask with Endowment of the MHWFAC (SH).

## Conflict of Interest

SH receives research grant support from Regeneron Pharmaceuticals.

The remaining authors declare that the research was conducted in the absence of any commercial or financial relationships that could be construed as a potential conflict of interest.
